# Effect of Flexible Sigmoidoscopy-Based Screening on Incidence and Mortality of Colorectal Cancer: A Systematic Review and Meta-Analysis of Randomized Controlled Trials

**DOI:** 10.1371/journal.pmed.1001352

**Published:** 2012-12-04

**Authors:** B. Joseph Elmunzer, Rodney A. Hayward, Philip S. Schoenfeld, Sameer D. Saini, Amar Deshpande, Akbar K. Waljee

**Affiliations:** 1 Division of Gastroenterology and Hepatology, Department of Medicine, University of Michigan Medical Center, Ann Arbor, Michigan, United States of America; 2 VA Ann Arbor Healthcare System and The Department of Internal Medicine, University of Michigan Medical Center, Ann Arbor, Michigan, United States of America; 3 Division of Gastroenterology, Department of Medicine, University of Miami Miller School of Medicine, Miami, Florida, United States of America; McGill University, Canada

## Abstract

**Background:**

Randomized controlled trials (RCTs) have yielded varying estimates of the benefit of flexible sigmoidoscopy (FS) screening for colorectal cancer (CRC). Our objective was to more precisely estimate the effect of FS-based screening on the incidence and mortality of CRC by performing a meta-analysis of published RCTs.

**Methods and Findings:**

Medline and Embase databases were searched for eligible articles published between 1966 and 28 May 2012. After screening 3,319 citations and 29 potentially relevant articles, two reviewers identified five RCTs evaluating the effect of FS screening on the incidence and mortality of CRC. The reviewers independently extracted relevant data; discrepancies were resolved by consensus. The quality of included studies was assessed using criteria set out by the Evidence-Based Gastroenterology Steering Group. Random effects meta-analysis was performed.

The five RCTs meeting eligibility criteria were determined to be of high methodologic quality and enrolled 416,159 total subjects. Four European studies compared FS to no screening and one study from the United States compared FS to usual care. By intention to treat analysis, FS-based screening was associated with an 18% relative risk reduction in the incidence of CRC (0.82, 95% CI 0.73–0.91, *p*<0.001, number needed to screen [NNS] to prevent one case of CRC = 361), a 33% reduction in the incidence of left-sided CRC (RR 0.67, 95% CI 0.59–0.76, *p*<0.001, NNS = 332), and a 28% reduction in the mortality of CRC (relative risk [RR] 0.72, 95% CI 0.65–0.80, *p*<0.001, NNS = 850). The efficacy estimate, the amount of benefit for those who actually adhered to the recommended treatment, suggested that FS screening reduced CRC incidence by 32% (*p*<0.001), and CRC-related mortality by 50% (*p*<0.001).

Limitations of this meta-analysis include heterogeneity in the design of the included trials, absence of studies from Africa, Asia, or South America, and lack of studies comparing FS with colonoscopy or stool-based testing.

**Conclusions:**

This meta-analysis of randomized controlled trials demonstrates that FS-based screening significantly reduces the incidence and mortality of colorectal cancer in average-risk patients.

*Please see later in the article for the Editors' Summary*

## Abstract 2

A systematic review and meta-analysis of randomized trials conducted by B. Joseph Elmunzer and colleagues reports that that flexible sigmoidoscopy-based screening reduces the incidence of colorectal cancer in average-risk patients, as compared to usual care or no screening.

## Editor's Summary

**Background:**

Colorectal cancer (CRC) is the second leading cause of cancer-related death in the United States. Regular CRC screening has been shown to reduce the risk of dying from CRC by 16%, and CRC screening can identify early stage cancers in otherwise healthy people, which allows for early treatment and management of the disease. Screening for colorectal cancer is frequently performed using a flexible sigmoidoscopy (FS), which is a thin, flexible tube with a tiny camera and light on the end, allowing a doctor to look at the inside wall of the bowel and remove any small growths or polyps. Although screening may detect early cancers, the life-saving and health benefits of screening are uncertain because the polyp may not necessarily progress. This could lead to anxiety and unnecessary interventions and treatments amongst those screened. Randomized controlled trials (RCTs) are needed to determine all of the risks involved in cancer screenings, however the guidelines that recommend FS-based screening do not rely upon RCT data. Recently, the results of four large-scale RCTs evaluating FS screening for CRC have been published. The conflicting results with respect to the incidence and mortality of CRC in these studies have called into question the effectiveness of endoscopic screening.

**Why Was This Study Done?:**

The results of RCTs measuring the risks and outcomes of CRC screening have shown varying estimates of the benefits of using FS screening. If better estimates of the risks and benefits of FS screening are developed, then the current CRC screening guidelines may be updated to reflect this new information. In this study, the authors show the results of a meta-analysis of published RCTs, which more precisely estimates the effects of FS-based screening on the incidence and mortality of colorectal cancer.

**What Did the Researchers Do and Find?:**

The researchers used the Medline and Embase databases to find relevant studies from 1966 to May 28, 2012. After screening 3,319 citations and 29 potentially relevant articles, five RCTs of high methodologic quality and 416,159 total subjects evaluating the effect of FS screening on the incidence and mortality of CRC were identified. The data were extracted and random effects meta-analysis was performed. The meta-analysis revealed that FS-based screening was associated with an 18% relative risk reduction in the incidence of CRC (0.82, 95% CI 0.73–0.91, *p*<0.001, number needed to screen (NNS) to prevent one case of CRC = 361), a 33% reduction in the incidence of left-sided CRC (RR 0.67, 95% CI 0.59–0.76, *p*<0.001, NNS = 332), and a 28% reduction in the mortality of CRC (RR 0.72, 95% CI 0.65–0.80, *p*<0.001, NNS = 850). The amount of benefit for those who adhered to the recommended treatment suggested that FS screening reduced CRC incidence by 32% (*p*<0.001), and CRC-related mortality by 50% (*p*<0.001).

**What Do These Findings Mean?:**

This meta-analysis of RCTs evaluating the effect of FS on CRC incidence and mortality demonstrates that a FS-based strategy for screening is very effective in reducing the incidence and mortality of CRC in patients. The current recommendations for endoscopic screening are based on observational studies, which may not accurately reflect the effect of FS-based screening on the incidence and mortality of CRC. Here, the authors performed a systematic review and meta-analysis of five recent RCTs to better estimate the true effect of FS-based screening on the incidence and mortality of CRC. Thus, the results of this meta-analysis may affect health policy, and directly impact patients and clinicians.

**Additional Information:**

Please access these Web sites via the online version of this summary at http://dx.doi.org/10.1371/journal.pmed.1001352.

- Cancer research UK provides comprehensive information about screening for colorectal cancers as does the UK National Screening Committee
- PubMed Health has general information about colon cancer
- The National Cancer Institute also has comprehensive resources on colorectal cancer and treatment
- The Mayo Clinic provides an overview of all aspects of colon cancer

## Introduction

Colorectal cancer (CRC) is the second leading cause of cancer-related death in the United States [1]. Endoscopic screening for CRC in average-risk patients has been widely adopted in the United States [2],[3] in accordance with multi-society and US Preventative Services Task Force (USPTF) guidelines [4],[5]. These recommendations for endoscopic screening, however, are based on observational studies [6]–[8], such as cohort and case-control studies, which have the potential to inaccurately estimate the true effect of this intervention on the incidence and mortality of CRC.

Since 2009, four large randomized controlled trials (RCTs) evaluating the effectiveness of flexible sigmoidoscopy (FS) for CRC screening in average-risk patients have been published [9]–[12]. While these RCTs were expected to provide strong evidence in support of existing screening guidelines, their results, when examined individually, have been inconclusive. Specifically, the effect of FS on CRC incidence and CRC-related mortality differs between studies.

We performed a systematic review and meta-analysis of RCTs to more precisely estimate the true effect of FS-based screening on the incidence and mortality of colorectal cancer in average-risk patients.

## Methods

### Literature Search

The study was conducted according to the PRISMA guidelines [13] (Text S1). A computer assisted search with the OVID interface to Medline and Embase was conducted to identify potentially relevant papers. A search for human studies published in these databases between 1966 and 28 May 2012 was performed using the exploded (exp) medical subject heading (MeSH) terms “(exp endoscopy OR exp sigmoidoscopy OR exp colonoscopy) AND (exp mass screening AND screening [keyword]) AND (exp colonic neoplasms OR exp colorectal neoplasms).” The search was augmented by manual searches of reference lists from potentially relevant papers to identify any additional studies that may have been missed using the computer-assisted strategy. Additionally, all abstracts from the American Gastroenterologic Association meetings (Digestive Diseases Week), American Society of Gastrointestinal Endoscopy meetings (Digestive Diseases Week), and United European Gastroenterology Week 2008–2011 were reviewed for potentially relevant abstracts. The search was not limited by language.

### Study Selection

Two investigators (BJE, AKW) independently reviewed titles and abstracts of all citations identified by the literature search. Potentially relevant studies were retrieved and selection criteria applied. The selection criteria were: (1) studies that examined the effect of FS screening on colorectal cancer; (2) studies that were prospective and randomized; (3) studies in humans; and (4) data not duplicated in another manuscript. Eligible articles were reviewed and data were abstracted in a duplicate and independent manner by two investigators (BJE, AKW).

### Data Extraction and Quality Assessment

The following data from selected studies were abstracted: year of publication, country in which study was conducted, age range of subjects, baseline CRC risk of subjects, endoscopic screening strategy, criteria used to trigger follow-up colonoscopy, duration of follow-up, number of subjects randomized to control and intervention groups, number of subjects undergoing index endoscopic evaluation, CRC incidence and mortality rates during the study period, and rates of follow-up endoscopic evaluation after the index screening. Discrepancies in data extraction were resolved by consensus.

The quality of included studies was assessed using criteria set forth by the Evidence-Based Gastroenterology Steering Group [14]. These criteria were: (1) concealed random allocation; (2) blinding of patients and caregivers; (3) equal use of co-interventions for treatment and placebo group; (4) complete follow-up of study patients; and (5) use of an intention to treat (ITT) analysis.

### Data Synthesis and Analysis

We conducted fixed and random effects meta-analyses using the metan command in the Stata 12.0 statistical package (Statacorp LP). The composite point estimates did not differ substantially between fixed and random effects models, therefore the random effects summary results are reported. We evaluated four endpoints: (1) the relative risk of CRC, (2) the relative risk of CRC-related death, (3) the relative risk of left-sided CRC, and (4) the relative risk of proximal CRC during follow-up. Proximal CRC was defined as CRC that developed proximal to the splenic flexure.

Many subjects randomized to endoscopic screening in these trials never received the screening. In addition, some subjects randomized to the control group did receive endoscopic screening. Therefore, in addition to examining the results of the ITT analyses of the component studies, we also performed a meta-analysis of the contamination-adjusted ITT results, which provides a more accurate estimate of the risk reduction achieved in those who actually adhered to the study treatment [15]. The contamination-adjusted ITT, also known as complier average causal effects, is favored over per-protocol analyses, as the latter may overestimate the benefit of endoscopic screening because patients who actually attend their flexible sigmoidoscopy (FS) are likely to possess other attributes or behaviors that are also protective against CRC, such as higher socioeconomic status, a healthier diet, and being generally more attentive to their health [15].

Since behaviors, including adherence to study assignment, are highly non-random, analyzing clinical trial results according to this behavior (which is exactly what occurs in per-protocol analysis) eliminates the benefits of randomization. The contamination-adjusted ITT provides a more accurate estimate of outcomes in those who actually adhere to the recommended intervention by treating randomization like an instrumental variable—a variable that determines the likelihood of exposure (screening versus no screening) but has no other direct or indirect effect on the outcome. The effect of the instrumental variable (randomization to treatment assignment) on the observed outcome can then be adjusted by the percentage of assigned participants who ultimately receive the treatment in order to yield the contamination-adjusted estimate.

For the study conducted in the United States, contamination rates were reported in both arms. For the European studies, it was assumed that no contamination occurred in the control group as routine endoscopic CRC screening was generally not conducted in these countries during the respective study periods.

## Results

### Literature Search

**Figure 1:**
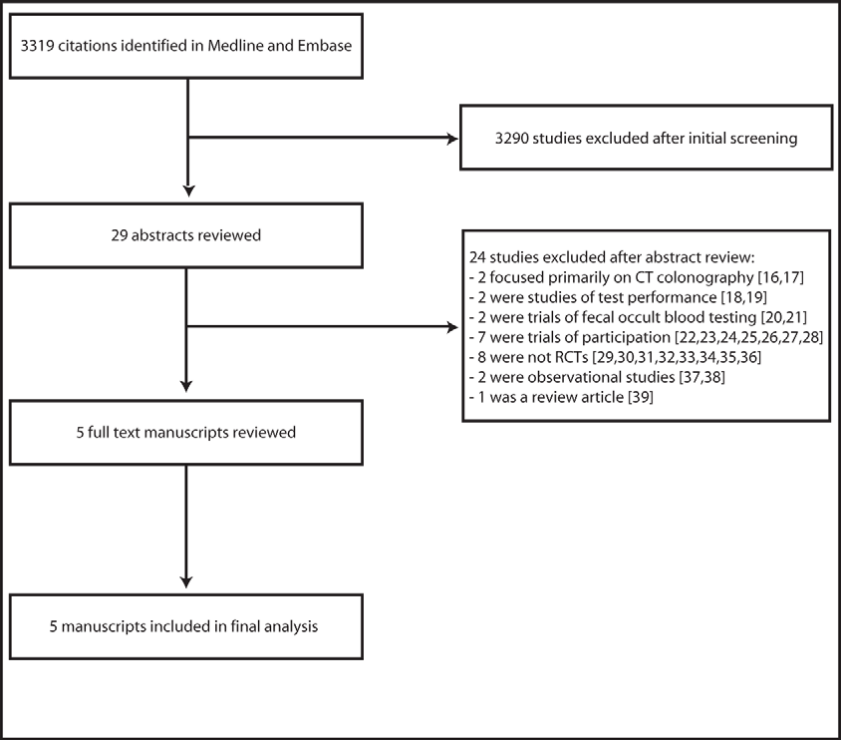
The literature search and selection process.

Searches of the Medline and Embase databases and bibliographies of relevant manuscripts yielded 3,319 citations and 29 potentially relevant articles. Abstract and brief manuscript review of these 29 articles resulted in five studies appropriate for detailed review, all five of which were included in the meta-analysis. The remaining 24 articles were excluded because they did meet inclusion criteria [16]–[39]. Specifically, eight studies were excluded because they were not randomized trials and seven were excluded because they were trials of participation, not long term outcomes. Two studies were excluded because they were observational studies of colonoscopy; two studies focused primarily on CT colonography, and two additional studies were excluded because they were trials of fecal occult blood testing. Two studies were excluded because they were comparative studies of test performance at index screening, and one was excluded because it was a review article. Scientific meeting abstract review yielded one additional potentially relevant study, which was excluded because it was not an RCT. There was 100% agreement between reviewers regarding study selection. A flow diagram depicting the search and selection process is provided in Figure 1.

### Characteristics of Included Studies

The five RCTs meeting eligibility criteria included 166,049 patients randomized to FS-based screening and 250,100 randomized to the control group (no recommended intervention). Four of the five studies demonstrated a statistically significant reduction in CRC incidence as a result of FS screening but only two of the five studies demonstrated a significant reduction in CRC mortality associated with FS-based screening. Characteristics of the five RCTs are listed in Table 1.

**Table 1:** Characteristics of included studies.

Characteristics	Shoen et al. 2012 (US) [11]	Segnan et al. 2011 (Italy) [12]	Atkin et al. 2010 (UK) [9]	Hoff et al. 2009 (Norway) [10]	Thiis-Evensen et al. 1999 (Norway) [40]
Screening strategy	FS at baseline, and another screening 3 or 5 y later. Patients with findings on FS were referred to their primary physician for follow-up.	Once-only lifetime FS with polypectomy of diminutive polyps^a^; full colonoscopy surveillance for patients with high-risk findings.	Once-only lifetime FS and polypectomy of small polyps^b^; full colonoscopy for patients with high-risk endoscopic findings.	FS with or without fecal occult blood testing; full colonoscopy with polypectomy for adenomatous polyps or any polyp >10 mm.	FS; full colonoscopy surveillance for patients with polyps.
Follow-up duration (median)	11.9 y	10.5 y for incidence, 11.4 for death	11.2 y	7 y for incidence, 6 y for death	13 y
*n* in the control arm	77,455	17,144	113,195	41,913	400
*n* in the screening arm	77,445	17,148	57,237	13,823	399
*n* attended screening	64,653	9,911	40,621	8,846	324
*n* developed CRC in control arm	1,287	306	1,818	362	10
*n* developed CRC in screening arm	1,012	251	706	123	2
Effect of FS on CRC incidence (ITT)	21% reduction (95% CI 0.72–0.85)	18% reduction (95% CI 0.69–0.96)	23% reduction (95% CI 0.70–0.84)	No difference	80% reduction (95% CI 0.03–0.95)
*n* died from CRC in control arm	341	83	538	99	3
*n* died from CRC in screening arm	252	65	189	24	1
Effect of FS on CRC mortality (ITT)	26% reduction (95% CI 0.63–0.87)	22% non-significant reduction (95% CI 0.56–1.08)	31% reduction (95% CI 0.59–0.82).	27% non-significant reduction (95% CI 0.47–1.13)	33% non-significant reduction (95% CI 0.03–3.19)

a ≤5 mm.

b ≤10 mm.

The earliest RCT of endoscopic screening for colorectal cancer was conducted in Norway as a proof of principle demonstration and published in 1999 [40]. Average-risk patients aged 50–59 y were randomized to no intervention versus FS, with subsequent enrollment into a colonoscopy with polypectomy surveillance program if polyps were detected during the index procedure. During a median of 13 y follow-up, two of 400 subjects (0.5%) randomized to the endoscopy group developed CRC compared with ten of 399 subjects (2.5%) randomized to the control group (*p* = 0.02).

Published in 2009, a second RCT from Norway reported the results of almost 60,000 average-risk patients between 55–64 y of age who were randomized to FS with or without fecal occult blood testing (*n* = 13,823) or no intervention (*n = *41,913) [10]. Patients with adenomatous polyps (or any polyp >10 mm) were referred for full colonoscopy. Subjects were followed for a median of 7 y. In the ITT analysis, no statistically significant reduction in colorectal cancer incidence or mortality was demonstrated.

The third study from the United Kingdom, published in 2010, randomized average-risk patients between the ages of 55–64 y to once-only FS and polypectomy for small polyps (followed by full colonoscopy in patients with high-risk endoscopic findings) or no intervention [9]. Subjects were followed for a median of 11.2 y. The ITT analysis of this study revealed that the incidence of CRC in the intervention group was reduced by 23% and CRC-related mortality was reduced by 31%.

Another European study, published in 2011, compared once-only FS with polypectomy (and follow-up colonoscopy for those with high-risk findings) with no screening in an Italian population of 34,292 average-risk patients aged 55–64 [12]. Subjects were followed for a median of 10.5 y for CRC incidence and 11.4 y for mortality. In the ITT analysis of this study, endoscopic screening was associated with an 18% reduction in CRC incidence and a 22% reduction in mortality, although this survival benefit was not statistically significant.

The most recently published study, conducted in the United States, randomized 154,900 mostly average-risk subjects, between the age of 55–74 y, to screening with FS (with a repeat screening at 3 or 5 y), or to usual care [11]. Subjects were followed for a median of 11.9 y. The authors report statistically significant benefits in the incidence and mortality of CRC associated with randomization to the screening group.

There were important differences in the conduct of the five included studies. Three of the studies (UK, Italy, US) enrolled and randomized patients who had previously indicated an interest in screening, whereas the other two studies randomized patients who had not previously been contacted by the investigators. In the US trial, both the screening and control groups had consented to enrollment and were aware of their involvement in the study, whereas in the other trials, the control group was unaware of its involvement.

The UK, Italy, and Norway 1999 studies evaluated a “once-only” FS strategy (no additional screening in those with a negative index examination) whereas the US study included a second interval FS. The Norway 2009 study included fecal occult blood testing in half of the subjects randomized to FS. In two studies (Norway 2009 and Italy), a colonoscope was used to perform FS, whereas a sigmoidoscope was used in the others.

In the UK and Italy studies, small polyps were resected endoscopically and patients were referred for full colonoscopy only when high-risk endoscopic features were detected on the index screen (e.g., polyp 1 cm or larger, three or more adenomas, and tubulovillous or villous histology). In the Norwegian studies, polypectomy was not performed during the index FS and all patients with an adenoma or other high-risk findings were referred for colonoscopy. In the US study, patients were referred back to the primary care physician for all decisions regarding follow-up endoscopic evaluation. Accordingly, the rates of follow-up colonoscopy differed between studies: 22% in the US study, 21% in Norway 2009, 5% in the UK study, and 4% in the Italian study (Norway 1999, not reported).

Despite these design differences, the primary objective of all these studies was clearly to determine the effect of FS on the incidence and mortality of CRC, and therefore we felt that a meta-analysis of the included trials was justified.

### Meta-analysis Results

**Figure 2:**
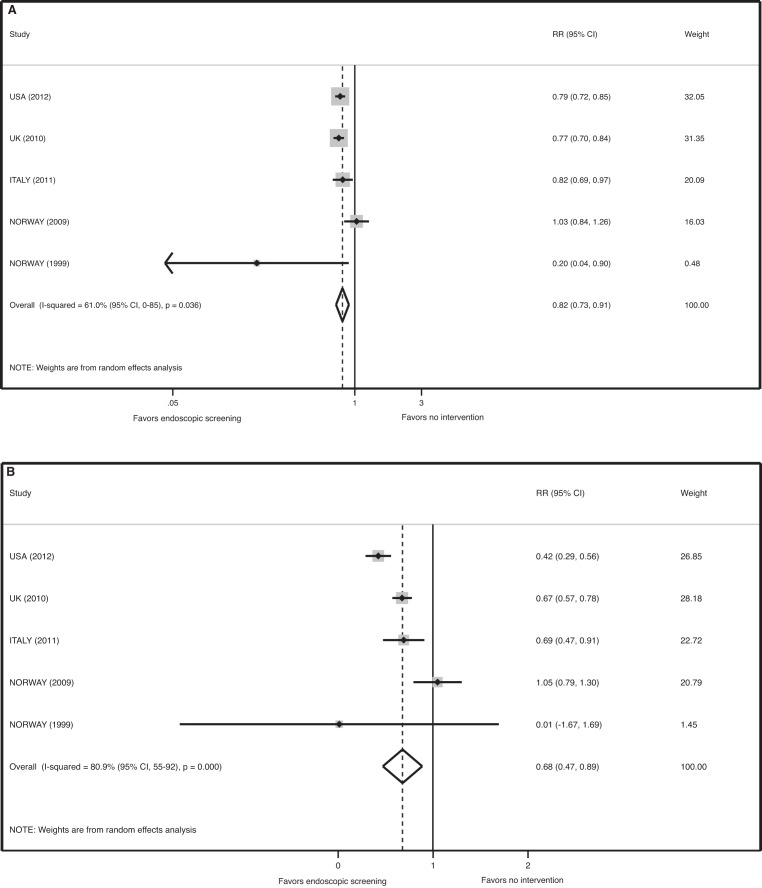
Meta-analysis of the effect of endoscopic screening on the incidence of colorectal cancer. (A) Pooled relative risk of ITT analyses. (B) Pooled relative risk of efficacy estimates.

Pooled analysis of the effect of FS screening on the incidence of colorectal cancer found significant heterogeneity between studies (*p = *0.036, I-squared = 61.0% [95% CI 0–85]). Meta-analysis of all five studies using a random effects model demonstrates that FS reduces the incidence of CRC by 18% by ITT analysis (relative risk [RR] 0.82, 95% CI 0.73–0.91, *p*<0.001) and by 32% in the efficacy analysis (RR 0.68, 95% CI 0.47–0.89, *p*<0.001) (Figure 2). The number needed to screen to prevent one case of CRC by ITT analysis is 361 (avoided cases per 1,000 = 2.8 [CI 1.4–4.0]).

Sensitivity analysis revealed that the Norway 2009 study is the source of statistical heterogeneity in the meta-analysis for the endpoint of CRC incidence. When this outlier study is removed, there was no evidence of heterogeneity in the four remaining studies (*p = *0.303, I-squared = 17.9% [95% CI 0–87]) and a meta-analysis of these four trials demonstrated a statistically significant reduction in the incidence of CRC associated with FS screening (RR 0.78, 95% CI 0.73–0.84, *p*<0.001 for the ITT analysis, and RR 0.58, 95% CI 0.41–0.76, *p*<0.001 for the efficacy estimate). For this analysis, the number needed to screen to prevent one case of CRC by ITT analysis is 278 (avoided cases per 1,000 = 3.6 [CI 2.7–4.4]).

**Figure 3:**
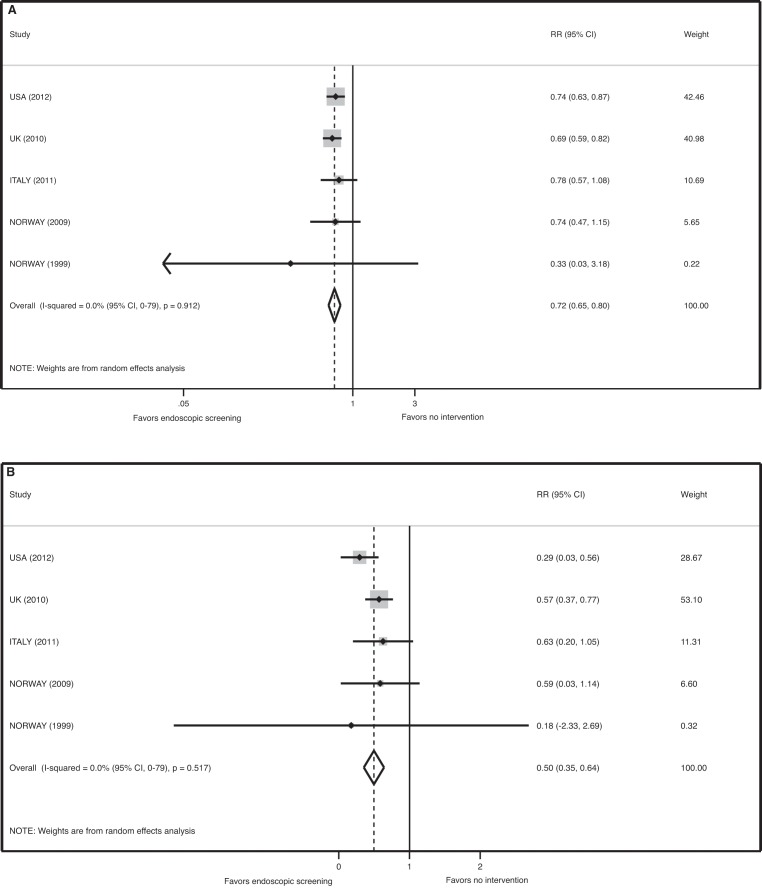
Meta-analysis of the effect of endoscopic screening on colorectal cancer mortality. (A) Pooled relative risk of ITT analyses. (B) Pooled relative risk of efficacy estimates.

There was no evidence of significant heterogeneity in the five RCTs for the endpoint of CRC mortality (*p* = 0.91, I-squared = 0.0% [95% CI 0–79]). The pooled relative risk for CRC mortality associated with FS screening was 0.72 (95% CI 0.65–0.80, *p*<0.001) for the ITT analysis and 0.50 (95% CI 0.35–0.64, *p*<0.001) for the efficacy estimate (Figure 3). The number needed to screen to prevent one death from CRC by ITT analysis is 850 (avoided deaths per 1,000 = 1.2 [CI 0.8–1.5]).

There was no evidence of significant heterogeneity for the endpoint of left-sided CRC (*p* = 0.1, I-squared = 49.0% [95% CI 0–81]). The pooled relative risk for the incidence of left-sided CRC in the screening arm was 0.67 (95% CI 0.59–0.76, *p*<0.001) for the ITT analysis. The number needed to screen to prevent one left-sided CRC by ITT analysis is 332 (avoided cases per 1,000 = 3.0 (CI 2.2–3.7)).

There was evidence of significant heterogeneity among the five RCTs for the endpoint proximal (non left-sided) CRC (*p*<0.001, I-squared = 86.0% [95% CI 69–94]). The random effects meta-analysis revealed a pooled relative risk for the incidence of right-sided CRC in the screening arm was 1.00 (95% CI 0.80–1.36, *p = *0.75).

### Assessment of Study Quality and Publication Bias

Quality assessment of the component studies revealed that all included studies had a score of 3 or 4, with each losing one point due to the inability to blind subjects and investigators. None of the studies had any “fatal” flaws for a randomized controlled trial.

The funnel plot asymmetry test for publication bias was negative using both the Begg's test *p = *0.743 (Kendall's tau) and the Egger's test *p = *0.515 (slope) *p = *0.256 (bias).

## Discussion

This meta-analysis of randomized controlled trials suggests that randomization to FS-based screening can reduce the risk of death from colorectal cancer by approximately 28%, and by 50% in those who actually receive screening. These findings provide grade A evidence for the inclusion of FS in CRC screening guidelines, akin to the justification afforded to fecal occult blood testing by prior RCTs [41]–[43].

In addition, meta-analysis showed that randomization to FS reduces the incidence of CRC by 18%, and by 32% in those who actually undergo this examination. The five included studies, however, demonstrated significant statistical heterogeneity for the endpoint of incidence. This heterogeneity may have been due to several design differences among the studies, including duration of follow-up, which was much shorter in the Norway 2009 study. Because CRC is a slow to develop malignancy, the benefits of screening are not immediate, but accrue over many years, and therefore this study is likely to have underestimated the reduction in CRC incidence because of inadequate follow-up duration. Because only five studies are included in this meta-analysis, however, the CI surrounding the I^2^ estimate of heterogeneity is very wide and definitive conclusions about heterogeneity cannot be made. Nevertheless, when the outlier study is removed from the meta-analysis, there was no evidence of heterogeneity in the four remaining studies (*p = *0.303), and a meta-analysis of these four trials demonstrated a statistically significant 22% reduction in the incidence of CRC associated with FS screening for the ITT analysis, and a statistically significant 42% reduction in incidence for the efficacy estimate.

When considering the results of this meta-analysis, both the ITT and efficacy estimates are informative, in that the ITT estimate is more important for the public health perspective (the maximum number of cancers and deaths that may be prevented by a population-based screening initiative) and the efficacy estimate is more appropriate when informing patients about the likely amount of individual benefit they may derive from screening. In this meta-analysis, both estimates suggest that endoscopic screening is effective in preventing CRC and CRC-related deaths.

This meta-analysis demonstrates that the protective effect of FS screening on the incidence of CRC is limited to the left side of the colon. Indeed, there appeared to be no reduction in proximal CRC as a result of FS-based screening. This finding may be due to the obvious disadvantage of FS—it only leads to screening of the right side of the colon if a left-sided adenoma or cancer is detected. Moreover, population studies to date have reported conflicting results regarding the benefit of full colonoscopy in reducing right-sided colon cancer. It remains unclear [44]–[46] whether this possible lack of incremental benefit is due to non-modifiable factors such as the biology of these tumors (more aggressive progression) or due to actionable deficiencies in screening colonoscopy performance (training and skill of endoscopist). While a small RCT of screening colonoscopy revealed a non-statistically significant 23% reduction in overall CRC incidence [47], large-scale randomized trials are eagerly awaited to allow accurate estimation of the effect of full colonoscopy on right-sided cancer. Three such studies are underway, however results may not be available for another 9–14 y [48]–[50].

An advantage of FS with polypectomy is that it appears to be an effective, low-cost, one-time intervention in the large majority of patients [9],[12], and is therefore particularly suitable for delivery in middle- and low-resource nations and to those with health care access barriers in the United States. Indeed, FS has been successfully delivered to uninsured patients in a “health fair” setting using the medical philanthropy platform [51]. Additional studies evaluating the feasibility of widespread delivery of health fair-style FS screening, including through medical philanthropic platforms, are necessary.

The results of this meta-analysis should be interpreted in the context of several important limitations. First, the design of the included studies differed in several substantive ways, ranging from the populations enrolled, to the screening strategy, to the duration of follow-up. While these differences may account for the varying results of the individual studies, they do not significantly detract from the primary objective of the RCTs, which was to assess the effectiveness of FS-based screening for CRC. Second, there was significant statistical heterogeneity for the endpoint of incidence. We believe that the most mechanistically plausible explanation for this heterogeneity is the short follow-up duration of the Norway 2009 trial; however, because of the small number of included studies, other possible contributing factors could not be formally explored using meta-regression.

This meta-analysis is further limited by the absence of studies from Asia, Africa, and South America, and therefore the results may not be generalizable to these parts of the world. Additional data regarding the burden of CRC and the utility of screening in these continents are necessary. Lastly, this meta-analysis focuses on the effect of FS as the index procedure, whereas full colonoscopy is the most widely employed screening strategy in the United States. Additional comparative effectiveness research of FS, colonoscopy, and stool-based testing is necessary to determine the most clinically and cost-effective CRC screening modality.

In summary, a meta-analysis of randomized controlled trials of endoscopic screening for CRC demonstrates that a FS-based strategy appears highly effective in reducing the incidence and mortality of this malignancy in average-risk patients.

## Supporting Information

Text S1 **PRISMA checklist.**
(DOC)

## Abbreviations

CRC: colorectal cancer
FS: flexible sigmoidoscopy
ITT: intention to treat analysis
NNS: number needed to screen
RCT: randomized controlled trial
